# Case Hepatic Endometriosis: A Continuing Diagnostic Dilemma

**DOI:** 10.1155/2009/407206

**Published:** 2009-07-05

**Authors:** P. J. Goldsmith, N. Ahmad, D. Dasgupta, J. Campbell, J. A. Guthrie, J. P. A. Lodge

**Affiliations:** ^1^Hepatobiliary and Transplant Unit, St James's University Hospital, Leeds LS9 7TF, UK; ^2^Department of Obstetrics and Gynaecology, St James's University Hospital, Leeds LS9 7TF, UK; ^3^Department of Radiology, St James's University Hospital, Leeds LS9 7TF, UK

## Abstract

*Background*. Intraparenchymal endometriosis of liver is rare. It may present as liver tumour and the diagnosis is not usually established till after surgery. *Case Outline*. A 48-year-old postmenopausal woman presented with right upper quadrant pain and a cystic liver mass. Liver function tests and tumour markers (*α*FP, CEA, CA 19-9, and CA 125) were normal. Radiological imaging (USS, CT and MRI) suggested a thick walled cystic mass involving segments IV and VIII with complex intracystic septations. Frozen section at operation suggested a benign cystadenoma. The cyst was enucleated using a CUSA (Cavitron ultrasonic aspirator). The final histology confirmed endometriosis. *Discussion*. Eleven cases of hepatic endometrioma have been reported and only four in postmenopausal women. Preoperative diagnosis poses a challenge and so far none of the cases have been diagnosed preoperatively. Surgery remains the treatment of choice. Accurate diagnosis at time of operation may avoid extensive liver surgery and its associated morbidity.

## 1. Introduction

Hepatic endometriosis is a rare disorder characterized by the presence of ectopic endometrium in the liver. An extensive review of the published literature in English Language showed that only eleven cases have been previously reported. Of the cases reported four have been described in postmenopausal women and six had a previous history of endometriosis. We report a case of hepatic endometriosis in a postmenopausal woman who presented with right upper quadrant pain as her only symptom. Possible methods of pathogenesis are discussed and literature is reviewed. The features of this case and the evidence in literature suggest that hepatic endometrioma should be included in the differential diagnosis for a woman of any age presenting with a hepatic mass, with or without a history of pelvic endometriosis. However, preoperative diagnosis is difficult despite exhaustive investigations in the absence of characteristic clinical and radiological features.

## 2. Case History

**Figure 1:**
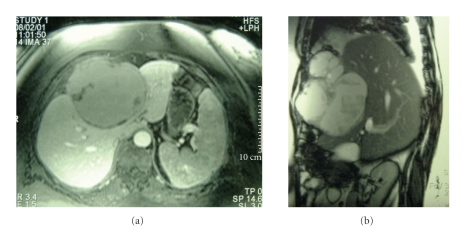
Magnetic resonance imaging (MRI) showing a cystic mass I segments IV and VIII, bulging into segments II and III and abutting the left and middle hepatic veins. The image also shows a soft tissue mass extending into the anterior abdominal wall.

A forty-eight-year-old nulliparous postmenopausal woman was referred to our unit for investigation of a cystic hepatic lesion. The patient gave a one and a half year history of relapsing and remitting chronic right upper quadrant pain requiring regular hospital admission and analgesia. There were no other significant symptoms. Four years previously she had undergone an abdominal hysterectomy and bilateral salpingo-oophorectomy for pelvic endometriosis. She had been on hormone replacement therapy (HRT) and had suffered no other gynaecological symptoms since. Her past history included hypertension and mild asthma. Examination revealed slight right upper quadrant tenderness but the patient's large body habitus precluded any accurate assessment for a mass. The tumour markers (*α*FP, CEA, CA 19-9, and CA 125) were within normal range as were the liver function tests. An abdominal ultrasound scan had previously shown a 9 × 11 cm cystic mass in segment IV of the liver. The wall appeared thick with complex septae, and there was apparent anterior extension into the extraperitoneal fat. Doppler studies indicated the mass to be completely avascular. Magnetic resonance imaging (MRI) showed an 11 × 13 cm cystic mass in segments IV and VIII, bulging into segments II and III and abutting the left and middle hepatic veins (Figures 1(a)  and 1(b)). The mass demonstrated was of high signal on both T1 and T2 images in keeping with hemorrhagic or mucinous contents. Incomplete septations were again demonstrated projecting into the lumen of the cyst with irregular nodularities of the wall. These features were consistent with a cystadenoma or cvstadenocarcinoma.

A laparotomy was performed with the intention of a left trisectionectomy (resection of segments 1, 2, 3, 4, 5, and 8). At operation the initial finding was that of a large cystic tumour in segment IV densely adherent to and infiltrating the abdominal wall and diaphragm. There was no evidence of metastases. The cyst was opened because it seemed unresectable and the interior was found to be thickly trabeculated with a ragged surface exudate. Biopsies were taken from the cyst wall and the septae. Frozen section histology suggested a benign cystadenoma. A nonanatomical resection was performed using a Cavitron Ultrasonic Aspirator (CUSA) and included segment IV and parts of segments II, III, and VIII. The tumour had to be peeled off from the left and middle hepatic veins. Some tumour was left on the diaphragm and pericardium as excision was considered unsafe. The patient made an uneventful recovery and was discharged home ten days postoperation. At followup she is asymptomatic 7 years after surgery with no evidence of recurrent disease.

**Figure 2:**
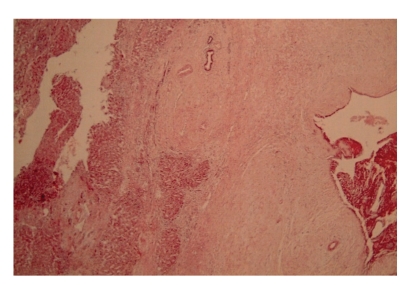
Low- power (X10, H + E) view of tumour capsule showing hepatocytes (on the left) and endometriotic epithelium with stroma (on the right). There are residual bile ducts entrapped within the capsule of the liver.

**Figure 3:**
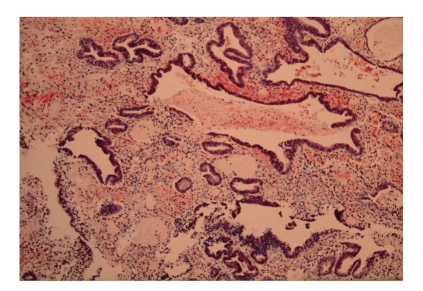
A high-power view (X25) of the endometriotic epithelium and stroma with haemosiderin laden macrophages.

Histological analysis showed an outer wall of fibrofatty tissue abutting large portal tracts with adjacent hepatic parenchyma showing no intrinsic abnormality. The inner wall was lined by fragments of endometrial tissue (Figure 2). Diaphragmatic sections also showed foci of endometriosis (Figure 3).

## 3. Discussion

Endometriosis, first described by Rokitansky in 1860 and is defined as the presence of endometrial tissue at sites other than the lining of the uterus [1]. The most common sites are within the pelvis (ovaries, fallopian tubes, uterosacral ligaments, and pouch of Douglas) though it has been described in nearly every part of the body excluding the spleen [2, 3]. Particularly unusual sites include the lungs, heart, pleura, diaphragm, thoracic cage, gastrointestinal tract, kidney, pancreas, bladder, umbilicus, bone, arms, legs, and scars [2]. To our knowledge there have only been eleven previously reported cases of hepatic endometrioma (Table 1) [1, 4–11]. 

The pathogenesis of endometriosis is uncertain. The most widely held theories are based upon possible mechanisms which transport endometrial tissue to distant sites in a fashion analogous to metastatic spread of neoplasm [12, 13]. The first postulated mechanism is of retrograde menstruation causing transcoelomic spread and implantation within the pelvis. This helps explain the predilection for gravity dependent pelvic deposits but fails to explain any distant or intraparenchymal lesion such as in this case. An alternative suggested mechanism is that endometrial tissue may reach distant places via the venous or lymphatic circulation. This would help explain both distant and intraparenchymal lesions, and it is this theory that has often found favour with previous reporters.

A different theory, that of coelomic metaplasia, suggests that peritoneal and submesothelial connective tissue have the potential to change to endometrial tissue [14]. This has so far been regarded as an unlikely explanation for hepatic endometrioma as the lesions of the liver are often completely intraparenchymal. Nine of the previously reported cases were completely intraparenchymal [1, 5, 8]. In this case, the pathology extended beyond the liver and there were adhesions to the diaphragm, rib cage, pericardium and abdominal wall. These adhesions may have been coincidental. Alternatively endometriosis may have started as coeclomic metaplasia of the overlying peritoneum spreading on the surface as well as growing into the liver, and eventually being “pinched off” as a liver lesion. Most of the reports including ours have shown hepatic endometrioma to occur in the left liver. It is suggested that the endometrial tissue may travel along the falciform ligament and seed around it [9].

Regardless of the pathogenesis, which remains controversial, this lady had a benign hepatic lesion which was not considered in the differential diagnosis. Indeed according to her ultrasound and MRI scans, the working diagnosis was that of a potentially malignant cystic neoplasm. In retrospect it appears that the patient did have a significant gynaecological history (pelvic endometriosis) for which she had undergone a hysterectomy and bilateral salpingoopherectomy. Of the previously reported cases a previous history of endometriosis has been reported [1, 8–11]. The remaining five had no previous gynaecological complaint [4–7]. Interestingly, none of the reported cases have described any cyclical exacerbation of symptoms coinciding with menstruation. Our patient was postmenopausal following a hysterectomy and bilateral salpingoopherectomy four years prior to presentation. She has been on hormone therapy (HRT). Whilst, it is unusual to develop and sustain denovo endometriosis in a postmenopausal woman with or without HRT, reactivation of endometriosis has been occasionally described in postmenopausal women on HRT [15]. Thus it seems that a significant gynaecological history may not be helpful in the preoperative diagnosis of hepatic endometrioma.

The ultrasound findings of a thick walled multiseptate cyst around the falciform ligament and high signals on TI and T2 sequence on MRI (suggesting presence of blood) should have raised the suspicion. But these findings are not specific. Inal et al. looked at the previous seven reported cases and concluded that there were no magnetic resonance, computer tomography, or ultrasound characteristics exclusively specific to hepatic endometrioma [10]. This is further complicated by the potentially fluctuating appearance of endometrial tissue under hormonal stimulation. An important question is whether a nonoperative method such as hormonal manipulation (Danazol and Gonadotrophin analogues, e.g., Buserelin, Goserelin, Leuprorelin) will be an effective sole treatment or prevent postoperative recurrence. There has been only one reported case of treatment with Danazol after the patient refused surgery [10]. However there is no followup data available to date. Our patient was also started on danazol. She is now 7 years following her resection and is symptom free. 

## 4. Conclusion

Hepatic endometrioma is a rare condition and its aetiology and pathogenesis are unclear. This is the twelfth reported case and it seems that no clues to its diagnosis can be gleaned from the history or examination and the imaging studies are not specific to this condition. For these reasons, hepatic endometrioma should be included in the differential diagnosis for a woman of any age presenting with a hepatic mass, with or without a history of pelvic endometriosis. Surgery remains the mainstay of treatment in patients with extensive or aggressive disease. However, in the case of young women fertility issues should be taken into consideration when considering treatment options. Frozen section histology at the time of surgery may help to avoid radical liver resection and its associated morbidity and mortality.

## Figures and Tables

**Table 1:** Features of reported cases of hepatic endometriosis.

Reference	Age (yrs)	Symptoms	Liver involvement	EH	Previous endometrial Tx	Treatment
Finkel et al. [4]	21	RUQ pain	Left lobe	No	Removal fallopian tube cyst	Cyst enucleation
Rovati et al. [5]	37	RUQ pain + mass	Left lobe	No	Non	Left lateral segmentectomy
Grabb et al. [6]	21	Epigastric pain	Left lobe	No	Removal of fallopian tube	Danazol + Deroofing
Verbeke et al. [7]	34	Acute abdominal pain	Right lobe	No	Non	Right hemihepetectomy
Verbeke et al. [7]	62	RUQ pain	Left lobe	No	Non	Segmentectomy
Gravello et al. [8]	34	Cyclical pain	Right lobe	Yes	Non	Metastectomy
Chung et al. [9]	40	Asymptomatic	Left lobe	Yes	Ovarian cystectomy	Segmentectomy
Inal et al. [10]	25	Pelvic pain	Right lobe	Yes	Medical tx for pelvic endometriosis	Danazol
Khan et al. [1]	31	Malaise, jaundice, abdominal distension	Bilobar	Yes	Hysterectomy and bilateral salpingo-oophrectomy	En bloc removal of right lobe mass, left lobe mass left.
Khan et al. [1]	59	RUQ pain + hepatomegaly	Right lobe	Yes	Removal of ruptured cyst	Right hepatectomy
Huang et al. [11]	56	Epigastric pain	Left lobe	Yes	Hysterectomy and bilateral salpingo-oophrectomy	Left lobectomy
Goldsmith et al. present case	48	Relapsing and remitting RUQ pain	Left lobe	Yes	Hysterectomy and bilateral salpingo-oophrectomy	Nonanatomical resection

EH: Endometrial history.
